# Praxeological Enactivism vs. Radical Enactivism: Reply to Hutto

**DOI:** 10.1007/s11097-022-09841-7

**Published:** 2022-08-17

**Authors:** Martin Weichold, Zuzanna Rucińska

**Affiliations:** 1 Institute of Philosophy, University of Regensburg, Universitätsstr. 31, D-93053 Regensburg, Germany; 2 Centre for Philosophical Psychology, University of Antwerp, Rodestraat 14, 2000 Antwerp, Belgium

## Abstract

In his recent paper “Getting Real About Pretense: A Radical Enactivist Proposal”, Daniel Hutto raises several objections against our so-called praxeological enactivist account of pretense (Weichold & Rucińska 2022). He argues that one should, instead, adopt his radical enactivist explanation of pretend play. In this short reply, we defend our praxeological enactivist account against his objections, and argue that it has crucial advantages over his radical enactivist alternative.

We are grateful to Hutto (2022, this volume) for discussing our praxeological enactivist account of pretense (Weichold & Rucińska 2022, this volume), and are happy to continue the conversation in this short reply. Hutto makes it clear that our praxeological enactivist (PE) account of pretense and his radical enactivist account (RE) share a lot. Still, he raises several objections against PE and presents RE as a more nuanced alternative. In what follows, we will defend PE against Hutto’s objections and show that it has crucial advantages over RE.

Hutto’s main objection is that PE is a “a solipsistic version of mind-dependent idealism” (Hutto, 2022). In reply, it is crucial to recall that PE aims to follow Varela, Thompson and Rosch (1991) in walking a middle way between realism and idealism. PE neither embraces a strong realism nor an idealism. The impression that it is idealistic might depend on one’s point of view. By analogy, if one is a die-hard conservative, everyone else on the political spectrum will appear to be a leftist. Likewise, if one is a strong realist, everyone else will appear to be an idealist. However, Varela et al. (1991) have, to our mind, provided good reasons for being skeptical of a too strong realism. At the same time, PE does not deny the undeniable. It can happily concede that there is a mind-independent reality. PE’s point is only that whenever living beings experience (that is, see, feel, smell, …) something, what they experience is the result of their making sense of objective surroundings. In contrast to what Hutto says, this does not mean to focus too strongly “on the organism side of the equation” (Hutto, 2022). Imagine two persons at the beach. One person perceives the beach as the most peaceful place in the world, while the other is annoyed by the scenery. How each of the persons perceives the beach depends on their individual sense-making. But this sense-making is always taking place in objective surroundings – e.g., the beach – which are then made sense of.^1^ For instance, the first person might perceive the beach as peaceful because she experiences pleasantly warm sunlight, soft sand, and soothing waves. The experience of the second person might, instead, be centered on unpleasant screams of seagulls. What is more, both persons’ dispositions for making sense of the beach at that particular time will have been shaped by their prior histories of interacting with beaches. Thus, what the two persons experience depends both on the objective surroundings they are in and on their dispositions for sense-making – which are themselves products of earlier interactions with objective surroundings. In sum, PE holds that experience consists neither in correctly representing objective states of affairs, as the realist assumes, nor in creating internal models, as the idealist thinks, but in making sense of one’s surroundings in embodied action. (We will explain this again with the example of pretending that a table is a lion’s cave below.)

As a consequence, PE argues that sense-making, or – as we call it – “understanding” and “interpretation” is ubiquitous. However, Hutto (2022) objects: “it seems quite possible for someone to engage in acts of pretending using the most minimal script-like knowledge and without any need for interpretation or understanding as these terms are standardly understood.” Following Wittgenstein, we are skeptical that there is one standard understanding of the terms “understanding” and “interpretation”. But for the sake of the argument, we can even agree that we do not use these terms in any standard way. Still, those terms, as *we* use them, are helpful for articulating the point we have just made: we human beings are always making sense of, or interpreting, our objective surroundings, and are then acting on our understood worlds. It is not an objective property of the sun that it is pleasantly warm. Rather, we interpret it as pleasantly warm. However, it is crucial to conceive of this “interpretation”, “understanding”, or “sense-making” in an appropriately de-intellectualized way, as it has been elaborated by hermeneutic phenomenology (Heidegger, 1927: §§ 31–34; Dreyfus 2005; see also Weichold 2015). Young children who are engaging in pretend play do not have to explicitly think of their pretense as pretense, nor do they have to “correctly understand” what it is they are doing. But they have to, for instance, interpret a block as a car or a pillow as a shovel. Even defenders of RE have to acknowledge this, if they want to allow for basic forms of pretend play that are more than mere automatic, reflex-like reactions.

Hutto makes a further objection that is related to the issue about idealism. In our paper, we discuss the example of a child who pretend plays being a lion in a lion’s cave. She is making sense of a certain object as her lion’s cave, namely an object that is, in ordinary social practices, interpreted as a kitchen table, that is, as a “piece of furniture providing a surface for eating” (Weichold & Rucińska 2022). In pretend play, the object is not interpreted as providing a surface for eating anymore. And in this sense, it ceases to be a kitchen table, thus understood. Hutto objects by saying that “the table is there throughout – whether the child is enjoying dinner with her family or playing lions” (Hutto, 2022). In other words, “[p]retending that a table is a battleship neither brings a battleship into being nor removes a table from being” (Hutto, 2022).

In reply, let us first make clear that, again, we do not want to deny the undeniable. There is an object the child is interacting with in her play. The object has a material existence. There are properties of the object that are not changed due to the pretend play: the object will still have the size of (say) 1.5 × 1.2 m and weigh 20 kg, when measured. It will be hard, when knocked on. However, our point is that the interpretation of the object as a kitchen table – as a surface for eating – is already an interpretation. It is an interpretation made from the perspective of a participant of the practice of, say, eating dinner. What is more, we might even call that object “a table” from our philosophical perspective of providing an analysis of pretend play, because from our perspective – the perspective of the philosophers interpreting child’s play – we interpret the object as a table that the child is pretending to be a lion’s cave. Our point is only that we human beings are *always* interpreting our surroundings – and not only in contexts of pretense. We interpret our surroundings differently when we engage in different practices. A chemist studying the properties of the table will interpret it as a collection of carbon isotopes; a carpenter engaging with the table will interpret it as a piece of great craftsmanship, perhaps focusing on its beautiful design, and philosophers can interpret it in numerous ways. Here we are essentially in agreement with ecological psychologists Ludger van Dijk and Erik Rietveld (2018) that the table must be looked at within a situation. Interpretation is ubiquitous, even though we are often not aware of it when it takes place in the context of ordinary practices.

Hutto’s own analogy to the duck-rabbit drawing is helpful in this regard. We would draw the analogy as follows: there are black lines on a white surface, or, respectively, an object that weighs 20 kg. From one perspective, the black lines are interpreted as a duck, and the object as a kitchen table. From another perspective, the black lines are interpreted as a rabbit, and the object as a lion’s cave. By contrast, RE appears to be committed to saying that *really* there is a duck or a kitchen table, and this duck or kitchen table is sometimes interpreted as a rabbit or a lion’s cave. This position sounds rather unattractive. Moreover, if RE adopted it, RE would have to face the “bypassing challenge”: how can a pretender “quarantine” the *true* meaning of an object in order to not confuse it with the *fictional* pretend meaning she projects onto the object? It becomes hard to solve this problem without appealing to mental representations. In our proposal, we do not face this challenge – there is no bypassing needed, because there is no *true* meaning to be bypassed in the first place (for an elaboration of this argument, see Rucińska 2019). What is more, it would be surprising if RE now adopted such a strong realism about the objects of everyday perception. For, in *Evolving Enactivism*, Daniel Hutto and Erik Myin propose themselves that perception is shaped by social practices (Hutto & Myin 2017: 175 f.). But then, they should be open to the thought that the mentioned object is perceived as a kitchen table only in the context of certain practices, such as eating.

While RE adopts a strong realism about the objects of everyday perception, it also embraces a strong *anti-*realism about pretense. This can be seen in Hutto’s next objection. He writes that “[t]o conceive of acts of pretense as inherently tied to the emergence of certain kinds of socio-cultural practices should make us immediately sceptical of the idea of finding the essence of pretense” (Hutto, 2022). Since PE analyzes pretense in terms of alternative sense-making that is related, in various ways, to ordinary practices, Hutto objects that “PE thus makes an essentializing move that RE rejects for a number of reasons” (Hutto, 2022). In reply, we first have to notice that it is unclear what is meant with “essence” and “essentializing move” here. In any case, PE does not offer a set of necessary *and sufficient* conditions for pretense. PE only provides necessary conditions by characterizing pretense as (1) an embodied activity that (2) consists of alternative sense-making which, in turn, (3) is related in various ways to ordinary practices. However, these conditions are not sufficient, and PE remains – in Wittgensteinean spirit – open to the observation that there is a great variety of different phenomena of pretense. Still, Hutto might object that providing those necessary, but not sufficient conditions is already an “essentializing move”. The question is, then, whether embracing such an essentialist view is a problem. There are clear cases where an essentialist view is problematic. For instance, entities like presidents or kings are constituted by social practices. They have no “essence”, in the sense that there are no intrinsic necessary conditions that make them what they are. A society is free to re-define what it means by “president”. By contrast, there are also cases where an essentialist view sounds reasonable. For instance, the corona-virus SARS-CoV-2 is not constituted by social practices. It does seem to have intrinsic necessary properties. Scientists can create models with predictive powers of it. Now, is pretense constituted by social practices, or is it not constituted by social practices and can have necessary conditions? PE opts for the second option. It is true that some activities like buying stock or marrying exist only inside social practices. But other activities like running, eating or sleeping are not constituted by social practices. According to PE, pretend play belongs to the second class. Even if a society did not have institutionalized practices of pretend play, young children would manifest pretend play: they would emulate the ordinary practices they observe in activities of alternative sense-making. Against this background, our approach leaves an important door open for very young children’s play, like teasing (see Reddy et al., 2022, this volume), or animal play, to be counted as pretense. RE does not seem to leave this door open.

This brings us to further advantages of PE over RE. Perfectly in line with Wittgenstein’s “Look, don’t think”-dictum, PE can acknowledge that the relationship between pretense and social practices is multifacetted – and much more complex than RE allows for. RE is right that *some* instances of pretend play take place in institutionalized social practices, like an institutionalized game of pretend playing knights. But there can also be instances of non-institutionalized, spontaneous and creative pretense, which can still, in one way or the other, emulate ordinary practices. For instance, a young child might observe the ordinary practice of baking bread, and then emulate this practice by pretend playing that her cushion is a dough (Szokolszky & Read 2022, this volume), without there being an established practice of pretend playing baking. PE emphasizes that there are many different degrees of the institutionalization of pretense, and many different ways how pretense can be related to ordinary social practices. What is more, by viewing pretend play as “practice practice” (Weichold & Rucińska 2022, this volume), PE can help to explain how young children learn to become participants in ordinary social practices. By contrast, RE’s account of pretense cannot explain this, since it assumes that pretense only exists in the form of social practices. On RE’s view, children must already master social practices in order to pretend. According PE, RE gets matters exactly backwards: it is more likely that pretend play helps children to learn to master social practices. This view is compatible with other ontogenetic and phylogenetic views that propose that pretense develops as an adaptive response for the purpose of cultural conformity (Bogdan, 2005) or acquisition of culture-specific skills, abilities, and knowledge (Adair & Carruthers, 2022).

In sum, in contrast to RE, PE steers a proper middle course between realism and idealism, avoids RE’s problematic strong realism about the objects of everyday perception and its problematic anti-realism about pretense, and shows that there is a great variety of ways in which pretense and ordinary social practices are related. If one wants to develop an enactivist account of pretense, there are good reasons for developing it in a *praxeological* enactivist and not in a *radical* enactivist way.

## Footnotes

1 Of course, “beach” is itself a human concept. Seeing something *as* a beach is then itself an interpretation. But that does not mean that the object that is interpreted as a beach does not really exist or is merely a projection.
